# Improved overall survival in patients developing endocrine toxicity during treatment with nivolumab for advanced non-small cell lung cancer in a prospective study

**DOI:** 10.1007/s40618-023-02268-0

**Published:** 2024-04-29

**Authors:** M. Albertelli, G. Rossi, E. Nazzari, C. Genova, F. Biello, E. Rijavec, M. G. Dal Bello, L. Patti, M. Tagliamento, G. Barletta, P. Morabito, M. Boschetti, A. Dotto, D. Campana, D. Ferone, F. Grossi

**Affiliations:** 1 Endocrinology Unit, IRCCS Ospedale Policlinico San Martino, Genova, Italy; 2 Endocrinology Unit, Department of Internal Medicine and Medical Specialties (DiMI), University of Genova, Viale Benedetto XV, 6, 16132 Genova, Italy; 3 Lung Cancer Unit, Department of Oncology, IRCCS Ospedale Policlinico San Martino, Genova, Italy; 4 Academic Oncology Unit, IRCCS Ospedale Policlinico San Martino, Genova, Italy; 5 Department of Internal Medicine and Medical Specialties (DiMI), Università Degli Studi di Genova, Genova, Italy; 6 Division of Oncology, Department of Translational Medicine, University of Piemonte Orientale, Novara, Italy; 7 UO Medical Oncology, IRCCS Azienda Ospedaliero-Universitaria di Bologna, Bologna, Italy; 8 Unit of Medical Oncology, Department of Medicine and Surgery, University of Insubria, ASST dei Sette Laghi, Varese, Italy

**Keywords:** Nivolumab, Hypothyroidism, Endocrine toxicity, NSCLC, Overall survival

## Abstract

**Purpose:**

Immune checkpoint inhibitors (ICPIs) disrupting PD-1/PD-L1 axis have revolutionized the management of advanced non-small cell lung cancer (NSCLC). Some studies identified the development of endocrine toxicity as predictor of better survival in cancer patients treated with ICPIs. The aim of study was to evaluate survival and new onset of immune-related endocrine adverse events (irAEs) in patients treated with nivolumab for advanced NSCLC.

**Methods:**

In a prospective study, 73 patients with previously treated advanced NSCLC received nivolumab in monotherapy. Blood samples were collected at each cycle to monitor thyroid autoimmunity, thyroid, adrenal and somatotroph axes, while thyroid morphology was evaluated by ultrasonography.

**Results:**

An impaired thyroid function was recorded in 23.4% of patients (*n* = 15). Eight patients developed asymptomatic transient thyrotoxicosis (ATT) evolving to hypothyroidism in 50% of cases. In addition, seven patients developed overt hypothyroidism without ATT and with negative autoantibodies. Patients who developed hypothyroidism proved to have better overall survival (OS) as compared with non-developers at both univariate (*p* = 0.021) and multivariate analyses (*p* = 0.023). The survival curve of patients with reduced IGF-I at baseline, or displaying its reduction during the follow-up, showed significantly reduced median survival compared to patients with normal/high IGF-I levels (*p* = 0.031).

**Conclusions:**

Thyroid function abnormalities are the major irAEs in patients treated with nivolumab, and hypothyroidism onset is associated with prolonged survival. Our findings indicate that the development of hypothyroidism is a positive predictive biomarker of nivolumab antitumor efficacy in patients with NSCLC. Low IGF-I levels could represent a negative prognostic factor during nivolumab therapy.

## Introduction

Immune checkpoint inhibitors (ICPIs) have radically changed the history of a wide spectrum of different neoplasms, including both solid and hematologic tumors [1–3].

Indeed, the recent history of non-small cell lung cancer (NSCLC) treatment has seen a dramatic turn with the introduction of ICPIs as well [4]. These agents exert their activity by blocking inhibitory signaling and enhancing T-cell activity against tumor cells. Moreover, these results have been obtained with tolerable toxicity profile, gaining them approval for numerous different types of cancer and paving the way for designing new trials in rarer ones [5, 6]. The first ICPI approved in the treatment of advanced NSCLC is nivolumab, an inhibitor of programmed cell death protein 1 (PD-1) [7]. The signaling cascade following the binding between PD-1 and its ligands (PD-L1 and PD-L2) is responsible for the inhibition of positive signaling, proliferation, and survival of peripheral tissue activated T cell, in order to prevent autoimmunity during inflammatory-mediated immune response. The same pathway is engaged by tumor cells to dampen early activation of the immune system and forestall tumor rejection [8].

Results from clinical trials with nivolumab in previously treated NSCLC have demonstrated an increase in median overall survival (OS) and overall response rate (ORR) in patients with pretreated non-squamous and squamous NSCLC, as well as an increase in median progression-free survival (PFS) in squamous histology [9, 10]. Nivolumab, together with the other ICPIs, shows great differences in term of frequency, types and grade of toxicities compared to chemotherapy. Due to their particular action on the immune system, these drugs present a significantly lower grade of hematologic toxicities, nausea, neuropathy, fatigue, and asthenia; however, a peculiar spectrum of adverse events (AEs) may be recorded. Indeed, their mechanism of action might result in impaired self-tolerance with subsequent development of immune-related AEs (irAEs). Previous experiences with ipilimumab (a CTLA-4 inhibitor) in advanced melanoma showed potential unconventional endocrine toxicities, and other data have been collected about nivolumab, although within ICPIs class some differences have been noticed [11, 12]. Compared with other irAEs caused by immune checkpoint blockade, endocrinopathies are unique because these manifestations are often irreversible. While hypophysitis is related to ipilimumab therapy with an estimated frequency around 11%, thyroid disfunction is the most frequent endocrine irAE reported during nivolumab treatment (hypothyroidism in 7% of cases) [13]. As far as nivolumab, results from Checkmate 057 and 017 trials, which explored the efficacy of nivolumab versus docetaxel in pretreated non-squamous and squamous NSCLC, reported up to 7 and 4% cases of hypothyroidism, respectively [9, 10].

Some recent evidence showed an improved overall survival in patients treated with ICPIs developing irAEs, suggesting that the onset of an autoimmune disease may be marker of a more potent immune activation consequent to the treatment, including the tumor-directed immune response [14–19].

In the last 5 years, after the arrival of nivolumab in pretreated NSCLC patients, new anti-PD-1 drugs have been added to the armamentarium of oncologists. Nowadays, all patients receive an ICPI in the first or in the second line of treatment. This prospective study was born at the dawn of the era of immunotherapy in NSCLC. The aim of this study was to prospectively evaluate the endocrinological toxicity of treatment with Nivolumab, the first of the available PD-1 antibodies, in the treatment of NSCLC. The long follow-up (> 60 months) allowed us to investigate the connection between toxicity and overall survival of patients in a real world setting.

## Patients and methods

Seventy-three patients (50 males, mean age 65, age range 44 –85 years) with advanced NSCLC who had previously been treated with at least one line of chemotherapy were enrolled in this single-institutional translational prospective research study, conducted by the Lung Cancer Unit and Endocrinology Unit of the IRCCS Ospedale Policlinico San Martino in Genova, Italy. The study was approved by the local ethical committee (CER Liguria, n° P.R. 191REG2015) and conducted within the Italian Expanded Access Program of nivolumab, which was open to previously treated patients with advanced NSCLC with both squamous and non-squamous histology. The consent has been obtained from each patient after full explanation of the purpose and nature of all procedures used. The enrolled patients received nivolumab at 3 mg/Kg every 14 days. The administration of nivolumab was continued until patient’s refusal, unacceptable toxicity, or confirmed and significant progressive disease (PD) in absence of clear clinical benefits, The pre-planned treatment duration in absence of progression was 2 years, after which the choice between treatment continuation or interruption was individually discussed with each patient; treatment beyond progression was allowed in case of persistent clinical benefit. In order to evaluate the impact of the treatment with nivolumab on the main endocrine axes, we collected and stored blood samples at baseline and at each cycle (every 14 days). Table 1 shows the hormonal assessment. Thyroid axis was explored in all 73 patients using TSH, fT3 and fT4. Thyroid morphology was evaluated by ultrasound examination at baseline in all patients (MyLab5, Esaote, Genova, Italy). For an adequate analysis of nivolumab-induced irAEs, patients with previous thyroid diseases at baseline were excluded. Thyroid irAEs were classified as grade 1 (G1), according to CTCAE v 4.0, when recorded as biochemical abnormalities in asymptomatic patients (asymptomatic transient thyrotoxicosis, ATT or subclinical hypothyroidism), and as G2 in the cases with typical hypothyroidism symptoms. ATT was defined as a clinical state of inappropriately elevated levels of circulating thyroid hormones (fT4 > 17.00 ng/L) and TSH < 0.270 mIU/L, detected for 4–6 weeks, which resolved spontaneously in asymptomatic patients. Subclinical hypothyroidism is defined by peripheral thyroid hormone levels within normal reference laboratory range but serum thyroid-stimulating hormone levels are mildly elevated (TSH > 4.200 mUI/L). We included “prior thyroid disease” as exclusion criteria to reduce possible bias.

**Table 1:** Evaluated hormones, acronym and normal range

Hormone	Abbreviation	Range
Thyroid-stimulating hormone	TSH	0.270–4.200 mIU/L
Free Triiodothyronine	fT3	1.80–4.60 ng/L
Free tetraiodothyronine	fT4	9.30–17.0 ng/L
Cortisol	Cortisol	3.70–19.40 µg/dL
Adreno cortico tropic hormone	ACTH	0.0–46.0 ng/L
Insulin-like growth factor 1	IGF-I	69–200 µg/L

Hypothalamus–pituitary–adrenal (HPA) axis was explored by measuring morning ACTH and cortisol levels in 55 patients. Finally, insulin-like growth factor 1 (IGF-I) was collected in all patients. All laboratory tests were performed at the San Martino central laboratory, Genoa, Italy. The clinical severity of patient with thyroid or adrenal dysfunction was graded using CTCAE 4.0 criteria. Patients’ characteristics are summarized in Table 2.

**Table 2:** Clinical characteristics of patients enrolled in the study

Clinical characteristics	*N*	%
Tot patients	73	100
Age (median)	65.6	
*Sex*		
Female	23	31.5
Male	50	68.5
*Smoking habit*		
Smoker	26	35.6
Former smoker	38	52.1
Never smoker	9	12.3
*Histology*		
ADK	58	79.5
Squamous	15	20.5
Stage IV	73	100
*Prior lines*		
1	30	41.1
2	20	27.4
> 3	23	31.5
*Dysthyroidism*		
*TSH* < 0.270 mIU/L	5	6.8
*TSH* > 4.200 mIU/L	4	5.5
*ECOG PS*		
0–1	65	89
2–3	8	11

### Statistical analysis

Categorical variables were expressed as percentage. Continuous variables were reported as median and range. Categorical variables were compared using Pearson Chi square or Fisher exact test, when appropriate. OS was defined as the interval between the start of the therapy and the time of death. OS was measured using the Kaplan–Meier method and the results were compared using the log-rank test. Predictive risk factors for OS were evaluated by univariate and multivariate analyses using the Cox proportional hazards method. Risk factors were expressed as hazard ratios (HR) and their 95% confidence interval (95% CI). The multivariate model was designed using the forward stepwise method after including all variables. All analyses carried out for predictive and risk factors are listed in the tables. The P value was considered significant when < 0.05. Statistical analysis was performed using a dedicated software (IBM—SPSS Statistics version 22).

## Results

### Endocrine evaluation

Endocrine evaluation planned at baseline was fully repeated at each cycle throughout the whole study. In our cohort of patients, immunotherapy discontinuation due to endocrine toxicities was never required. The specific assessed hormonal axes are discussed below.

#### Thyroid axis

Thyroid function was assessed in all patients (*n* = 73). At baseline, 9 patients (12.3%) had impaired thyroid function: 5 had reduced TSH levels (2 previous thyroidectomies with levothyroxine replacement therapy excess, 1 amiodarone-induced thyrotoxicosis and 2 subclinical hyperthyroidism with negative anti-TSH receptor antibodies [TRAb]) and 4 patients presented with increased TSH at baseline (1 previous thyroidectomy requiring hormone replacement therapy adjustment, 1 hypothyroidism with positive autoimmunity, 2 subclinical hypothyroidism with negative autoimmunity). These 9 patients, although fully monitored, were excluded from the final analysis of nivolumab-induced thyroid dysfunctions: total patients analyzed *n* = 64 (Table 3). Interestingly, thyroid profile was not substantially modified by therapy with nivolumab in this subgroup of patients, both in case of hyper- or hypofunctionality.

**Table 3:** Clinical characteristics of analyzed patients in the study

	With irreversible hypothyroidism		Without irreversible hypothyroidism		*p*
	*N*	%	*N*	%	
*Sex*					0.395
Female	5	22.7	17	77.3	
Male	6	14.3	36	85.7	
Age (median)	72		67		0.065
*Smoke*					0.056
Smoker	2	9.5	19	90.5	
Former smoker	6	16.7	30	83.3	
Never smoker	3	42.9	4	57.1	
*Histology*					0.723
Squamous	2	14.3	12	85.7	
ADK	9	18.4	40	81.6	
*ECOG PS*					0.241
0–1	11	19.0	47	81.0	
2–3	0	0	6	100	

During the follow-up, 8 out of 64 patients (12.5%) developed asymptomatic transient thyrotoxicosis (ATT), evolving to hypothyroidism in 50% of cases (*n* = 4); only 3 of the 4 patients with thyrotoxicosis evolving to hypothyroidism showed increased autoantibodies levels (TPOAb and TgAb). Conversely, 7 different patients (11%) with negative thyroid autoimmunity developed hypothyroidism, without displaying transient thyrotoxicosis (Table 4).

**Table 4:** Frequencies and features of thyroid irAEs

Tot evaluable patients *n* = 64	ATT → Eu	ATT → Hypo	Hypo without ATT	Thyroid dysfunctions
Ab −	4	1	7	12 (80%)
Ab +	–	3	-	3 (20%)
Tot	4	4	7	15 (100%)
Final outcome	Eu (*n* = 4)	Hypo (*n* = 11)		

Hypo, Hypothyroidism; ATT, asymptomatic transient thyrotoxicosis; Eu, euthyroidism; Ab, antibodies; tot, total

The average FU in which thyroidal axis impairment was observed was 15 weeks (range 2–56) for isolated hypothyroidism and 4 weeks for transient thyrotoxicosis (range 1–10) that turned to hypothyroidism after a median of 12 weeks from the beginning of the anticancer treatment. Two patients with ATT were treated with low beta-blockers doses and the remaining 6 patients not required beta-blockers, steroid therapy or methimazole for thyroiditis. Replacement therapy with levothyroxine was necessary in only two cases of patients with hypothyroidism, since other patients presented with a subclinical hypothyroidism. Treatment of hypothyroidism, as well as thyrotoxicosis, was performed according to standard therapy guidelines [20].

In patients with subclinical G1 hypothyroidism, the median TSH level at the time of diagnosis was 11.66 mIU/L (range 6.2–47.1). In symptomatic G2 hypothyroidism, the median TSH was 55.5/34.88 mIU/L (range 47–64/30.3–64.4).

Overall, during the study, the patients developing thyroid disfunction as irAEs were 15 (23.4%), 11 cases (17%) of hypothyroidism and 4 with ATT evolving in euthyroidism (Tables 3, 4). None of them required suspension of nivolumab therapy due to thyroid disfunction.

As far as ultrasound findings, none of patients developing ATT that turned to hypothyroidism had evidence of thyroid nodule. Among patients with ATT with subsequent euthyroidism, 2 had evidence of thyroid nodules and 2 had an increased thyroid volume. In only one patient developing hypothyroidism ultrasound revealed thyroid nodules. Among patients with baseline hyperthyroidism, 3 showed thyroid nodules at ultrasound examination.

#### HPA axis

Data were available in 55 subjects, 14 of which were already taking steroid therapy at maximum dosage of 10 mg of prednisone. Among the remaining 31 patients, only 8 showed significant cortisol alterations: 2 with elevation in morning serum cortisol levels, 5 with reduction and one displaying alternate elevated or reduced cortisol levels. All these cortisol changes were not clinically relevant and were transient. No patients in our cohort displayed ACTH alterations.

#### GH-IGF-I axis

**Fig. 1:**
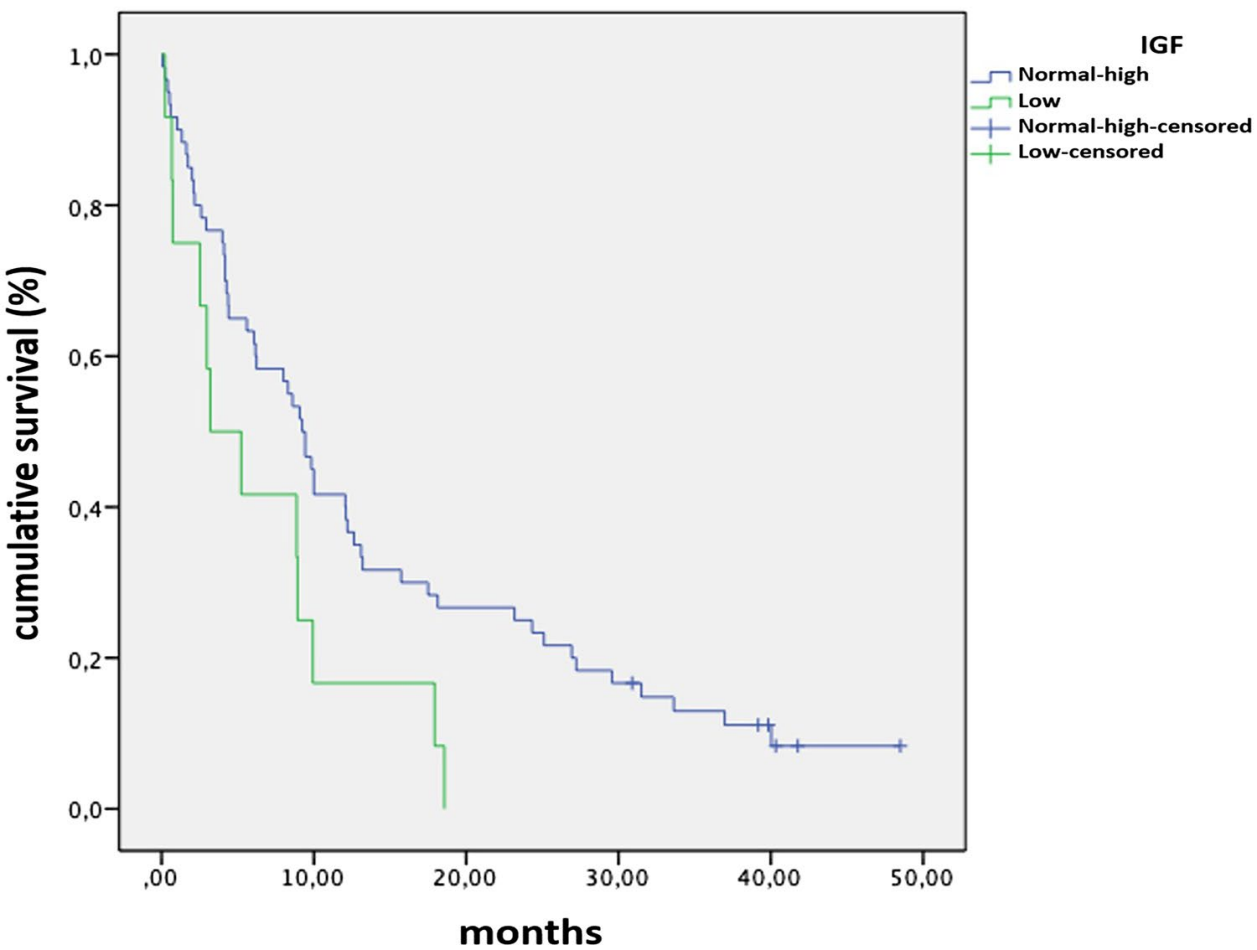
Survival analysis of patients according to IGF-I levels (*p* = 0.031)

From 73 patients, 36 cases reported at least one abnormal value. In particular, 24 patients showed elevated IGF-I levels, while 12 patients had significantly reduced IGF-I levels at baseline (8) or during therapy (4). In this series, patients with reduced IGF-I values at baseline, or with a reduction during follow-up, showed a significant reduction in median survival compared to the group of patients with normal and high IGF-I levels at Kaplan–Meier analysis (*p* = 0.031) [Fig. 1].

#### Overall survival and irAEs onset

All statistical analyses were performed excluding patients with previous impaired thyroid function (*n* = 9). Indeed, the characteristics of final group of the 64 patients, according to the presence or absence of hypothyroidism, are shown in Table 3. As previously said, a total of 15 patients developed thyroid irAEs, including 7 developing hypothyroidism and 8 developing thyrotoxicosis which resolved in either euthyroidism (*n* = 4) or hypothyroidism (*n* = 4).

Since in our population the main endocrine irAEs observed were thyroid-related, we considered this subgroup to perform the survival analysis in comparison with the subgroup of non-developers. Median follow-up of the patients was 9.1 months (range 1–20 months) starting from the day of the administration of the first nivolumab cycle.

At the comparison between hypothyroidism and non-hypothyroidism developers, no statistically significant difference was observed in the distribution of several features such as age, sex, histology, staging of the tumor and performance status (Table 3).

**Fig. 2:**
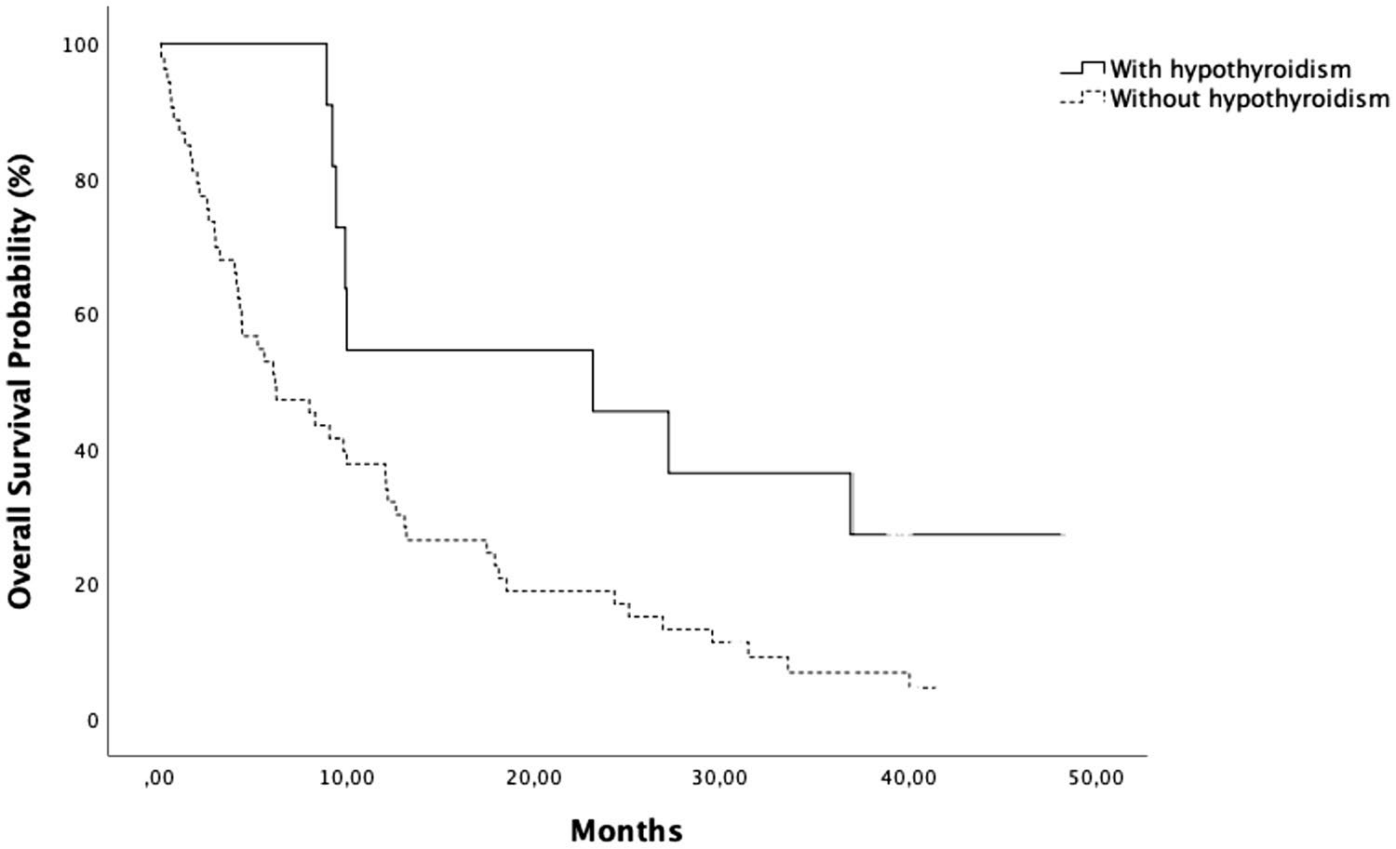
Survival analysis of patients according to development of irreversible hypothyroidism (*p* = 0.023)

At the univariate analysis, the median OS for the group of patients with hypothyroidism was 23.2 months, whereas the median OS of those without hypothyroidism was 6.2 months (*p* = 0.023). These results are confirmed at the multivariate analysis (*p* = 0.021) as shown in Table 5 and at Kaplan–Meier analysis (*p* = 0.023) represented in Fig. 2.

**Table 5:** Univariate and multivariate analyses

Characteristic	Univariate analysis			Multivariate analysis		
	HR	95% CI	*p*	HR	95% CI	*p*
Sex (male)	0.90	0.52–1.54	0.702			Ns
Age	1.01	0.98–1.04	0.496			Ns
Smoke	1.16	0.50–2.70	0.735			Ns
Squamous cell carcinoma	1.16	0.62–2.16	0.644			Ns
*Lines*						
> 2	1					Ns
2	1.31	0.72–2.37	0.376			Ns
1	0.71	0.35–1.14	0.336			Ns
**IGF low**	**2.11**	**1.04–4.26**	**0.037**	2.04	0.99–4.25	0.054
Low PS (2–3)	0.99	0.39–2.50	0.978			Ns
**Irreversible hypothyroidism**	**0.41**	**0.19–0.87**	**0.021**	**0.41**	**0.19–0.88**	

Bold values indicates statistically significant results

## Discussion

Pivotal studies with nivolumab in patients with non-squamous and squamous NSCLC reported, as endocrine AEs, only hypothyroidism in about 7% and 4% of patients, respectively [9, 10]. In line with the published data on endocrine irAEs associated with nivolumab, in our cohort, thyroid function abnormalities were confirmed to be the major endocrine irAE [21–25]. Conversely, in this cohort, we did not record any hypophysitis, confirming that this life-threatening event, in contrast with the evidence observed with CTLA-4 blockade, is rare during PD-1 inhibitors treatment [22–24, 26].

Development of impaired thyroid function in the present study was reported in 23.4% of patients (*n* = 15). Hypothyroidism seems to be the most frequent irAE and occurred in 17% of cases (*n* = 11), often without a concomitant increase in thyroid antibodies. This incidence of hypothyroidism was superior when compared to that reported in the registration trials [9, 10]. However, in these latter studies, the features of hypothyroidism and the presence of a previous ATT were not specified. The 6.2% of patients (*n* = 4) developed a transient thyrotoxicosis, evolving to hypothyroidism on autoimmune basis in 75% of cases. Another 6.2% of patients (*n* = 4) showed only a transient thyrotoxicosis with negative thyroid autoimmunity, which turned into euthyroidism without requiring any treatment. In these cases, since the ultrasound examination performed in this subgroup of patients revealed the presence of thyroid nodules or goiter, it is possible to postulate that even an excess of iodine intake due to contrast enhanced radiological exams (CT-scan every 4–8 weeks) could have played a role in the recorded transient thyroid abnormalities.

Considering the entire cohort of patients, no significant correlation was observed between functional thyroid abnormalities and ultrasound morphology.

Furthermore, even though the limited number of cases with previous and concomitant thyroid alterations in our trial (9/74), we did not record any effect of nivolumab on thyroid function in this subgroup of patients. Indeed, our results confirm that a preexisting thyroid alteration does not represent a major exclusion criterion for nivolumab therapy, if combined with appropriate management of thyroid disease, consistently with other publications [27]. We preferred to include “prior thyroid disease” as exclusion criteria to reduce possible bias. This may be seen as a limitation of the study, but it is aimed to assess the impact of therapy on the occurrence of endocrine adverse events. We know that this reduces the possibility of extending these results to the entire population.

As regards HPA, data are confounded by concomitant therapies known to interfere with this hormonal assessment. However, in our cohort, we did not identify any case of isolated ACTH deficiency induced by autoimmune-related mechanism, as recently reported in few cases with metastatic malignant melanoma or other cancers treated with nivolumab [28–34].

However, it is interesting to note that 32% of our patients presented with elevated IGF-I levels at baseline. This finding is in line with published data correlating high IGF-I serum levels to an increased risk of lung malignancy [35–37], as well as colon and breast cancer [38, 39], and showing that patients with lung cancer have significantly increased circulating IGF-I levels compared to healthy controls [40]. On the contrary, reduced IGF-I levels at baseline in specific settings of patients can be explained by the presence of advanced metastatic disease, while the increase in IGF-I levels during therapy may reflect the improvement of metabolic conditions and performance status [41–43]. Recent evidences in the literature, regarding other different neoplasms [43–45], have shown that lower levels of IGF-I were predictive of worse outcome. According to these observations, even in our study, IGF-I low levels were associated to shorter OS compared with patients with normal/high IGF-I. In fact, survival curve of patients with reduced IGF-I at baseline, or displaying a reduction during follow-up, showed a significantly reduced median survival compared to those of patients with normal/high IGF-I levels (*p* = 0.031). Therefore, low IGF-I at baseline, or a decrease of IGF-I levels during nivolumab treatment, could be considered as a negative prognostic factor in this setting of patients. We could speculate whether the reduction in IGF-I levels was secondary to treatment or disease progression and, due to the distribution of cases with low IGF-I at baseline and during therapy in our series, we could hypothesize that this phenomenon is more related to disease progression. Furthermore, it is known that IGF-I is an expression of the nutritional status and is related to the metabolic state of the patient; therefore, it could be considered as a surrogate of performance status (PS). In our series, interestingly, low IGF-I performed better than PS in predicting worst patient outcome, being significant in univariate (*p* = 0.037) and at the limits of significance in multivariate (*p* = 0.054; see Table 5). However, the size of our sample does not allow us to draw clear conclusions. Therefore, this observation needs to be further validated in a larger study cohort of patient with advanced NSCLC.

Finally, regarding the connection between OS and new onset of hypothyroidism as irAE, we observed that patients developing hypothyroidism showed a lower mortality rate as compared with non-developers. Median OS in the first subgroup of patients was 23.2 months, whereas in the second was 6.2 months (*p* = 0.023). Univariate and multivariate analyses confirm these data [Fig. 2 and Table 5].

The onset of endocrine irAEs has already proved to be a favorable response predictor to ICPIs therapy in recent studies in different cancers [18, 46–48], and such findings are even of older knowledge when regarding the treatment of melanoma with ICPIs, due probably to the greater experience acquired so far with tumor, for which ICPIs were first approved [10, 11]. When considering monotherapy with nivolumab in lung cancer, most of the studies available in the literature are retrospective [14, 17, 19, 49]. Many of them appear to confirm our findings: Baldini et al. and Ricciuti et al. [19, 49] found that the irAEs-developing group showed a substantial difference in OS as compared to non-developers (*p* < 0.0001 in both studies). Ricciuti et al. even observing that patients who developed ≥ 2 irAEs during treatment had a significantly longer median PFS and OS compared to those with one irAE. Campredon et al. [50] and Sbardella et al. [51] did not find statistical significance, still they observed a trend toward an improved OS in the group of patients developing AEs (*p* = 0.069 and *p* = 0.27, respectively), though the sample size was relatively small.

On the contrary, Ksienski et al. [52] observed a decreased survival in patients developing severe AEs, though the considered patients had to suspend treatment at very early stages (landmarks are 6 and 12 weeks).

Among few prospective studies [53–56], Kim’s group was the only one to specifically evaluate the association of thyroid dysfunction during PD-1 blockade with the treatment efficacy in patients with NSCLC. They observed a significantly longer OS for patients developing thyroid irAES as compared with the non-developers (*p* = 0.025). However, Kim et al. in their prospective trial enrolled patients received PD-1 blockade (nivolumab or pembrolizumab) without making a distinction in outcomes between the two drugs [57].

Our results, consistent with data of the vast majority of the cited papers, show significantly longer OS in patients who developed hypothyroidism as irAEs, suggesting that the dampening of PD-1 signaling pathway may be responsible for both endocrine toxicity and antitumor response, so that hypothyroidism may be seen as a marker of an enhanced therapeutic effect. Indeed, to the best of our knowledge, this represents the first prospective study proving a better OS in NSCLC patients developing hypothyroidism as irAES during treatment with nivolumab in monotherapy.

In conclusion, routine monitoring of thyroid function should be performed at baseline and after each cycle of nivolumab. Global pituitary function, including HPA axis, should be investigated and monitored in all case and, obviously, in case of the onset of signs and symptoms of hypophysitis or cortisol deficiency.

Reduced IGF-I levels in specific settings of patients can be explained by the presence of advanced disease and have shown to be predictive of worse outcome in our cohort.

Our findings regarding the connection between hypothyroidism onset and better OS seem to indicate that the development of hypothyroidism as irAE is a positive predictive biomarker of nivolumab antitumor efficacy in patients with NSCLC.

These results need to be confirmed by further studies. However, a close collaboration between oncologist and endocrinologist is of crucial importance to provide patients with the best care and avoid unnecessary therapy discontinuation.
